# Pairwise Analysis Can Account for Network Structures Arising from Spike-Timing Dependent Plasticity

**DOI:** 10.1371/journal.pcbi.1002906

**Published:** 2013-02-21

**Authors:** Baktash Babadi, L. F. Abbott

**Affiliations:** 1 Center for Theoretical Neuroscience, Department of Neuroscience, Columbia University, New York, New York, United States of America; 2 Swartz Program in Theoretical Neuroscience, Center for Brain Sciences, Harvard University, Cambridge, Massachusetts, United States; École Normale Supérieure, College de France, CNRS, France

## Abstract

Spike timing-dependent plasticity (STDP) modifies synaptic strengths based on timing information available locally at each synapse. Despite this, it induces global structures within a recurrently connected network. We study such structures both through simulations and by analyzing the effects of STDP on pair-wise interactions of neurons. We show how conventional STDP acts as a loop-eliminating mechanism and organizes neurons into in- and out-hubs. Loop-elimination increases when depression dominates and turns into loop-generation when potentiation dominates. STDP with a shifted temporal window such that coincident spikes cause depression enhances recurrent connections and functions as a strict buffering mechanism that maintains a roughly constant average firing rate. STDP with the opposite temporal shift functions as a loop eliminator at low rates and as a potent loop generator at higher rates. In general, studying pairwise interactions of neurons provides important insights about the structures that STDP can produce in large networks.

## Author Summary

The connectivity structure in neural networks reflects, at least in part, the long-term effects of synaptic plasticity mechanisms that underlie learning and memory. In one of the most widespread such mechanisms, spike-timing dependent plasticity (STDP), the temporal order of pre- and postsynaptic spiking across a synapse determines whether it is strengthened or weakened. Therefore, the synapses are modified solely based on local information through STDP. However, STDP can give rise to a variety of global connectivity structures in an interconnected neural network. Here, we provide an analytical framework that can predict the global structures that arise from STDP in such a network. The analytical technique we develop is actually quite simple, and involves the study of two interconnected neurons receiving inputs from their surrounding network. Following analytical calculations for a variety of different STDP models, we test and verify all our predictions through full network simulations. More importantly, the developed analytical tool will allow other researchers to figure out what arises from any other type of STDP in a network.

## Introduction

Spike timing-dependent plasticity (STDP) is a widespread mechanism that modifies synapses on the basis of the intervals between ensembles of pre- and postsynaptic spikes [1], [2]. The most prevalent form of STDP involves potentiation of the synapse when presynaptic spikes precede postsynaptic spikes, and depression for the reverse ordering [3]. STDP is inherently a local synaptic modification rule because the determinant of synaptic modification is the timing of pre- and postsynaptic spikes. Neurons, on the other hand, are typically embedded in interconnected networks in which each neuron receives thousands of synapses from other neurons [4], [5]. A number of studies have explored how STDP shapes the distribution of synaptic weights for a population of synapses converging onto a single neuron [6]–[10]. Here, we consider the more difficult problem of bridging the gap between the locality of STDP and the global structures that it generates in a recurrent network of spiking neurons.

The problem of STDP in a recurrent network has been addressed before in a number of studies [11]–[22]. The generally antisymmetric shape of the STDP window, in which reversing the ordering of pre- and postsynaptic spikes reverses the direction of synaptic change, led to the proposal that this synaptic modification rule should eliminate strong recurrent connections between neurons [11], [23]. This idea has recently been expanded by Kozloski and Cecchi [21] to larger polysynaptic loops in the case of “balanced” STDP in which the magnitudes of potentiation and depression are equal. These authors also showed that balanced STDP organizes network neurons into in- and out-hubs. The possibility of enhancing recurrent connections through pair-based STDP was also proposed by Song and Abbott [11] and is further explored by Clopath and colleagues [22] in a more complex model. An excessively active group of neurons has been shown to decouple from the rest of the network through STDP [13], and in presence of axonal delays, STDP enhances recurrent connections when the neurons fire in a tonic irregular mode [14]. Here, we show that, surprisingly, all of these network properties can be explained through an understanding of the effect of STDP on pairwise interactions of neurons. This provides an analytically tractable way of relating the structures arising in a network to properties of the STDP model being used to modify synapses.

## Results

STDP is characterized by a change of synaptic strength, 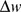, induced by a pair of pre- and postsynaptic action potentials with time difference (pairing interval) 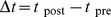. The functional relation between the synaptic modification and the pairing interval is given by
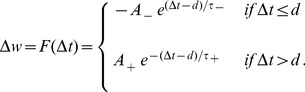
(1)


The positive parameters 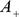 and 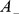 specify the maximum potentiation and depression, respectively. We express the synaptic strengths in units of the membrane potential (mV), so 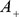 and 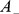 have mV units. The time constants 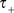 and 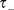 determine the temporal spread of the STDP window for potentiation and depression. The parameter 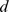, when it is nonzero, introduces a shift in the STDP window such that for positive values of 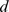 even in cases where a presynaptic action potential precedes a postsynaptic spike by a short interval (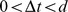), the corresponding synapse gets depressed [10]. Conversely, for negative values of 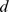 the synapse gets potentiated even when a postsynaptic action potential precedes a presynaptic spike by a short interval (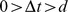) [14]. We recover conventional STDP by setting 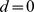. For conventional STDP, spike interactions are all-to-all, meaning that all possible pre-post pairs contribute to plasticity. However, using an STDP model with only nearest-neighbor interactions does not qualitatively alter the obtained results (see Text S1). The main motivation for using all-to-all interactions in the first cases analyzed below is the clarity of explaining the resulting synaptic dynamics with these interactions. In the case of shifted STDP (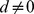), we use only nearest-neighbor spike pairs to drive plasticity, for reasons of stability explained in [10]. Moreover, as the results show, the background firing rates of neurons play an important role in synaptic dynamics with nearest-neighbor interactions if the STDP window is shifted.

The effect of STDP on synaptic weights depends not only on properties of the STDP window function of equation 1, but also on how boundaries are imposed on the range of allowed synaptic weights [7], [9], [24]. In the case of “soft” boundaries, the synaptic dynamics will be confined only to a narrow region in the middle of the range of synaptic strengths [24] (see Text S1, figure S2), whereas in the case of “hard boundaries”, the synapses can explore the whole range of their allowed strengths. Therefore, we restrict our analysis to the case of hard boundaries as it results in more interesting network structures.

**Figure 1:**
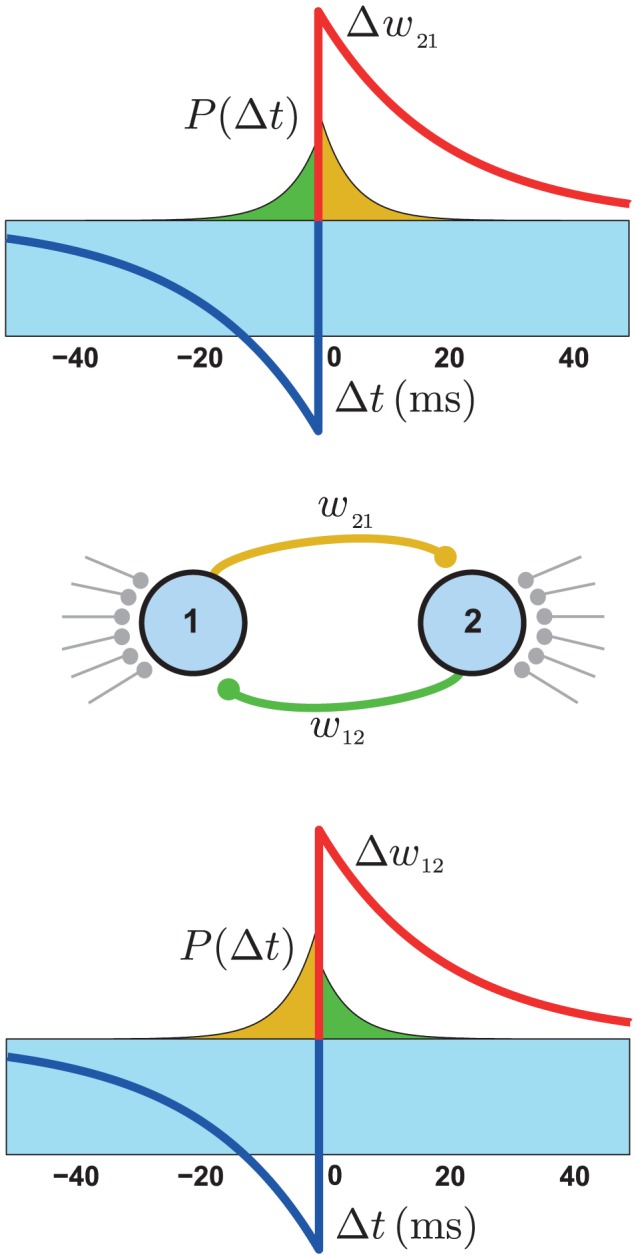
Pairwise interactions of neurons through reciprocal synapses and the corresponding spike-train cross correlations. Two representative neurons embedded in a network are shown with their reciprocal synapses (middle). 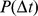 is the probability density of pairing intervals 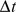 between the two spike trains, which is superimposed on the STDP window. Both synapses are modified by the pairings of the baseline spike trains of the neurons (blue area in top and bottom panels). Whenever neuron 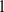 fires, the synapse 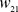 (yellow) induces a transient increase in the firing rate of neuron 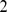 which in turn increases the probability of pairings with short intervals (yellow areas in top and bottom panels). This transient firing rate increase potentiates 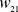, because it falls into the potentiation domain of STDP (top), but it falls into depression domain for 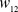 (bottom). The transient increase in the firing rate of neuron 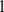 in response to spikes of neuron 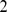 (green areas in top and bottom panels) has the opposite effect.

To gain analytical insights into the structures that arise from STDP in a network and then to verify those insights, we take a dual approach for each form of STDP we study. First, we consider a pair of connected neurons that we imagine to have been extracted from a full network, representing the remaining neurons of the network as independent Poisson input to each of these neurons (figure 1, middle, with network inputs shown as gray connections). This simplified model allows us to perform a detailed analytic study of how STDP affects the synapses between the two explicitly modeled neurons (drawn in yellow and green in figure 1, middle). Modifications of synapses from the rest of the “network” onto these two neuron is not modeled directly, but is duplicated by changing the mean effective network input into each neuron, which alters its baseline firing rate. Despite these simplifications, many of the structures induced by STDP in a large network can be explained by analogy with properties of this two-neuron system. As the second component of our approach, we verify that analytic predictions extracted from the simplified model apply to full networks with STDP acting at all synapses by simulating these full networks.

The two representative excitatory neurons drawn from the network are labeled neuron 1 and neuron 2, and are reciprocally connected (figure 1, middle). We denote the strength of the synapse from neuron 1 to neuron 2 as 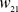 and the strength of the synapse from 2 to 1 as 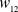. Due to the additional “network” inputs, each neuron fires at a baseline rate, given by 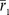 and 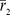, respectively. We assume that there are no significant correlations between the baseline spike trains of neurons 1 and 2, because the recurrent inhibition is strong. In the presence of a strong recurrent inhibition, spontaneous fluctuations in the activity of excitatory and inhibitory populations accurately track each other and cancel the effect of shared input [25], and this is true for the networks we study except where otherwise indicated (figure S3). It is worth noting that we do not assume that the baseline firing rates are constant; they are functions of the synaptic strengths in the network and therefore change with STDP. We are interested in the local dynamics of pairs of reciprocal synapses given the current values of baseline firing rates. As we will see below, this pairwise analysis can predict how the baseline firing rates will change over time as STDP affects the network. Under the conditions we assume, synapses are modified primarily by random pre-post pairings of their baseline spike trains. The average amount of modification due to this baseline activity is the same for both synapses (i.e. the same for both 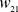 and 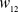). On top of the baseline firing, the reciprocal synaptic connections induce correlations between the spike trains of the two neurons. Each spike arriving from neuron 1 to neuron 2 transiently increases the firing rate of neuron 2 proportional to 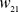 (figure 1, yellow areas). This transient increase (or causal bump) potentiates 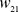 (figure 1, top) and depresses 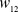 (figure 1, bottom). Likewise, the causal bump in the cross-correlation induced by neuron 2 into neuron 1 (figure 1, green areas) potentiates 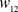 and depresses 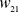. Taken together, the average drift of the synaptic pair can be expressed as
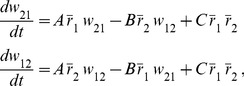
(2)where the coefficients 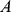, 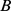 and 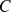 can be calculated from the parameters of the neuronal and plasticity models (see Text S1). In each equation, the coefficient 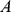 represents the potentiation induced on a synaptic weight by the causal effect it has on the firing of its postsynaptic neuron, 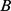 represents the depression induced in the same synapse by the causal effect of its reciprocal synapse on presynaptic firing, and 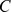 characterizes the synaptic modification due to random pairings of the baseline spike trains of the two neurons. Because the synaptic strengths are bounded between 0 and 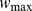, the drift of the synaptic pair, as described by these equations, is restricted to a limited region in the state space . As we will see in the following sections, this restriction results in a number of interesting effects that would not arise in a strictly linear system.

In what follows, we first examine the effect of different parameterizations of the STDP window on the synaptic pair. This leads to a number of predictions about the structures that arise from STDP in networks. We then test each prediction through numerical simulations of a large network. In the case in which the STDP window is not shifted (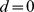), the time constants of the window are assumed to be equal (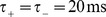), but we vary the balance between potentiation and depression by changing the maximum values 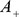 and 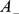. The same qualitative results hold when the maximum values are set equal and the potentiation/depression balance is modified by changing the time constants (see Text S1).

### Balanced STDP

**Figure 2:**
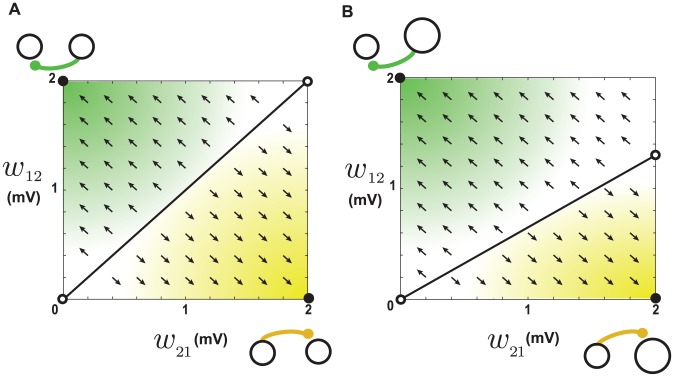
Dynamics of reciprocal synapses when STDP is balanced. **A.** When the baseline firing rates of the two neurons are the same (20 Hz), synapses that have the same weight remain at equilibrium (solid diagonal line). When one of the synapses is initially larger than the other, it grows while the smaller one shrinks until they hit the boundaries and eventually settle into the attractors at the bottom right or top left. Attractors are depicted as filled circles and unstable fixed points as open circles. The attractors correspond to unidirectional connections as depicted schematically. The arrows show the trajectories of the synapses due to plasticity, obtained by numerical evaluation of equation (2) at each point. **B.** When the baseline firing rates are not the same (20 Hz vs. 30 Hz), the line of equilibria becomes tilted. The neuron with the lower rate (depicted smaller) is more likely to receive a unidirectional synapse.

**Figure 3:**
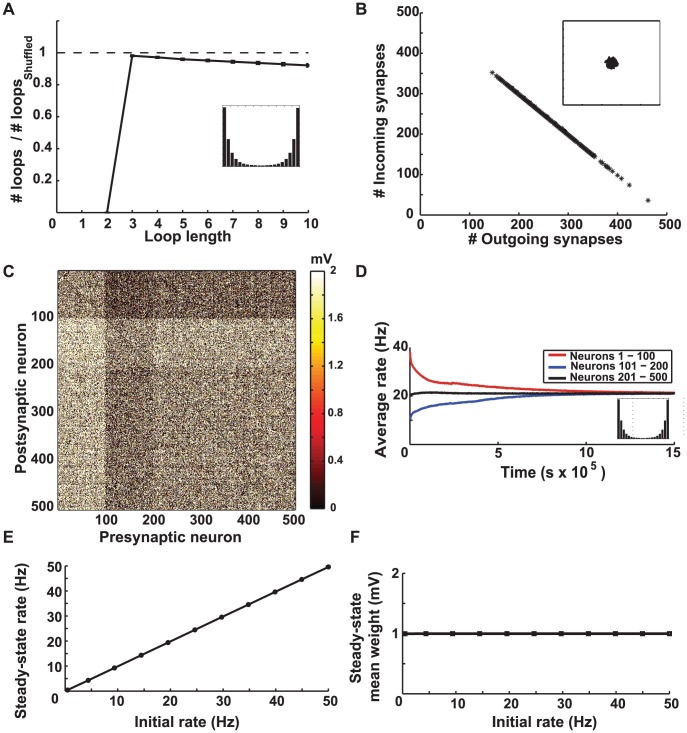
Simulation results for a network with balanced STDP. **A.** The number of loops in the steady-state weight matrix, divided by the number of loops in a shuffled version of this matrix, as a function of the length of the loop. The initial firing rate of the network was 20 Hz. Error bars depict the standard deviations from using 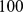 different shuffled versions. The ratios are less than one (dashed line) for all loop lengths, so STDP decrease the number of loops from the chance level. The inset shows the final distribution of weights in the network. **B.** The number of above-threshold incoming synapses plotted against the number of above-threshold outgoing synapses in the steady-state connectivity matrix when the initial rate was 20 Hz. Each mark depicts a neuron. The threshold values 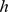 for counting the loops is equal to the mean weight (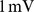). Inset shows the same plot for a shuffled version of the steady-state weight matrix. **C.** The steady-state weight matrix when neurons 1–100 receive stronger external input and initially fire at 40 Hz, neurons 101–200 receive weaker external input and initially fire at 10 Hz, and the rest of the neurons fire initially at 20 Hz. Neurons 1–100 receive fewer synapses from the network (dark horizontal band on top) and send out more synapses (bright vertical band on the left), so they have turned into out-hubs. Neurons 101–200 receive more synapses from the network (bright horizontal band) and send out less synapses (dark vertical band), so they have turned into in-hubs. **D.** The average firing rate of the three sub-populations of neurons as a function of time. The firing rates tend to equalize at steady-state. The inset shows the final distribution of weights in the network. **E.** The average steady-state and initial average firing rates of the network are equal. **F.** The steady-state mean synaptic weight as a function of the initial rate. It remains constant in the middle of the allowed range (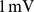) regardless of the initial rate and distribution of weights. Note that in **E** and **F** the statistics of the external inputs is the same for all neurons, hence the average initial rates are the same, unlike in **C** and **D**.

The simplest form of STDP that we consider is balanced with equal potentiation and depression domains, i.e. with 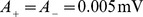. In this case, the coefficient 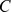 vanishes because the baseline potentiation and depression cancel each other. In addition, the coefficients 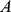 and 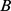 are equal. These conditions greatly simplify the system of equations (2). We visualize the dynamics of the synaptic pair by phase planes (figure 2). Note that all phase planes throughout this paper are snapshots of the dynamics for a given network firing rate, and as we will see below, they should be recomputed if the baseline firing rates change appreciably. When the baseline firing rates are equal (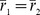), the values of the synaptic weights do not change when 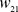 and 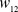 are equal, i.e. the synaptic drift is zero on the line 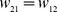 (figure 2A, solid line). However, this equilibrium is unstable. If the two synapses have unequal strengths, the stronger synapse grows even stronger and weakens the other synapse until they reach the boundary of their allowed range (figure 2A, arrows). Then, the synapses continue their dynamics along the boundary edge until they reach the upper-left (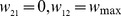) or lower-right (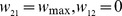) corner of the state space (figure 2A, filled circles), depending on which synapse was stronger to begin with. These “attractors” of the synaptic dynamics indicate that, at steady-state, loops between pairs of neurons are eliminated by this form of STDP. A linear system of differential equations cannot have more than one attractor. The existence of two attractors here is a consequence of restricting the dynamics to a limited range, and it suggests that STDP favors unidirectional connections and eliminates loops in a network (figure 3A), in agreement with the results of [21]. The attractors also imply that, for each neuron, the strengthening of an incoming synapse is accompanied by the weakening of an outgoing synapse and vice versa. If we consider the effect of this interplay at the level of a network, it is expected that for each neuron the number of above-threshold incoming and outgoing synapses will be linearly related with a coefficient of -1, such that their sum remains constant. Our simulation results and previous work [21] also confirm this prediction (figure 3B).

When the baseline rates of the two neurons are not equal, the line of equilibrium is tilted to 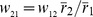 (figure 2B, see Text S1). As a result, the size of the basins of the two attractors differ, and the outgoing synapses of the neuron with the higher firing rate are more likely to strengthen, while its incoming synapse are likely to weaken (figure 2B, top-left corner). Conversely, outgoing synapses from the neuron with the lower firing rate are more likely to weaken and its incoming synapses strengthen. If we generalize this behavior to the context of a network, an important prediction can be made: neurons with low initial firing rates should attract strong excitatory synapses onto themselves but project weaker synapse to other neurons. Neurons with high firing rates should experience the opposite trend; they lose incoming synaptic input through synaptic weakening, while their outgoing synapses strengthen. Therefore, if the external input is biased to give a sub-population of excitatory neurons an initially higher (lower) firing rate than the rest of the network, these neurons will become out-hubs (in-hubs) through STDP. We tested this by setting the mean of the external input to the neurons such that the initial firing rate of the first hundred excitatory neurons (1–100) was 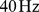, the initial firing rate of the next hundred excitatory neurons (101–200) was 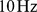, and the initial firing rate for the rest of the excitatory neurons (201–500) together with that of the inhibitory neurons was 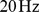. The results show that the sub-population with high initial rate indeed turns into out-hubs, while the sub-population with low initial rate turns into in-hubs once the synaptic weights reach steady state (figure 3C).

Another related prediction is that the firing rates of neurons in the network tend to equalize through STDP. This is because neurons with high initial firing rate become out-hubs, thereby receiving less input from the rest of the network, which lowers their final, equilibrium firing rates. At the same time, they share their initial high firing rate with the other neurons of the network through the strengthening of their outgoing synapses. The opposite happens to neurons with low initial firing rates; they turn into in-hubs. As a result the final firing rates of the neurons become homogenized across the network at steady-state. To test this prediction, we tracked the evolution of the average firing rates of the above three sub-populations throughout the simulation. The results confirm that the final firing rates of all three sub-populations equalize once the synaptic weights reach steady-state (figure 3D).

As another prediction, the steady-state mean of the synaptic weights is expected to converge to the midpoint of its allowed range, regardless of the initial distribution of weights and the initial firing rate of the network. This is due to the precise balance between the potentiation and depression domains of STDP in this case. If the initial mean is already at the midpoint of the allowed range, the potentiation and depression events have equal probabilities across the network due to this balance. If the initial mean is smaller than the midpoint, a number of depression events would not be fully realized because they are likely to push the synaptic weights to 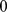, so equality of potentiation and depression is disrupted in favor the former, and the mean tends to increases. Similarly, if the initial mean is larger than the midpoint, some of the potentiation events push the corresponding weight above the maximum value and are truncated. This decreases the mean. Therefore, the mean tends to the midpoint in both cases, and the baseline firing rate (which already has the tendency to equalize) does not change this scenario. The simulation results confirm this prediction (figure 3F). If the initial value of the weights are drawn from a uniform distribution, the mean will be at the midpoint from the very beginning, and it remains there throughout the simulation, making the final network firing rate equal to the initial rate (figure 3E). Note that in figure 3E, and in similar figures to follow, we plot quantities as a function of the initial firing rate of the network, as opposed, for example, the external input used to modify this rate. We do this to make apparent changes in the network firing rate caused by STDP.

### STDP with dominant potentiation

In studies of a single neuron receiving Poisson input through synapses that are modified by STDP, it has been shown that stability requires depression to dominate over potentiation [6]–[9]. If potentiation dominates in this case, all the synaptic weights get potentiated to the maximum allowed value. Interestingly, for STDP within a network of neurons, potentiation dominated STDP can be stable because this instability is counteracted by the depression induced on reciprocal pairs of synapses. In other words, if one synapse between a reciprocally connected pair of neurons grows, the other synapse is likely to be weakened, preventing the outcome in which all the synapses are maximally potentiated.

**Figure 4:**
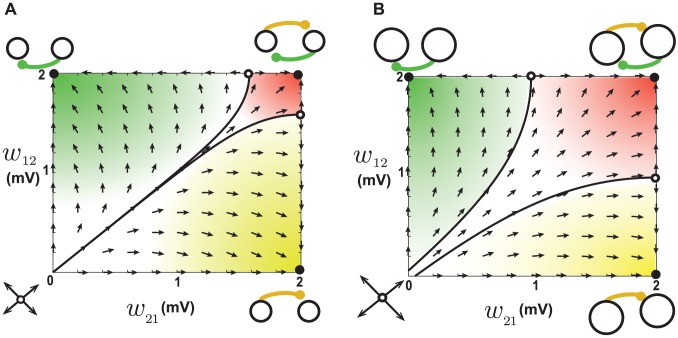
Dynamics of reciprocal synapses when STDP is potentiation dominated. **A.** When the baseline firing rates of the two neurons are both 20 Hz, an unstable fixed point exists out of the allowed range, schematically illustrated at the bottom left. Arrows show that the trajectories drift away from this outlying fixed point. Initial conditions starting within the red area end up at the attractor at the top-right corner, which corresponds to recurrent connections. Trajectories that hit the boundaries perpendicularly delineate the borders of the basins of attraction (solid curves). Initial conditions in the yellow area end up at the attractor at the bottom right, corresponding to a unidirectional connection from neuron 1 to neuron 2. Initial conditions within the green area go to the attractor at top left, corresponding to a unidirectional connection from neuron 2 to neuron 1. **B.** The same as **A** when the baseline firing rates are 50 Hz. The basin of attraction for recurrent connections (red area) becomes larger when the baseline firing rate increases.

When the potentiation/depression balance is tipped in favor of potentiation (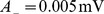 and 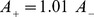 in our examples), the coefficient 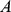 in equation (2) becomes larger than 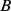 (see Text S1). In addition, the baseline parameter 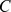 is positive. By setting the right-hand-sides of equations (2) to zero, the fixed point values of the two synaptic weights are found to be 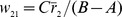 and 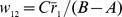. Both of these values are negative, so the fixed point lies outside the allowed range of synaptic strengths. Furthermore, this fixed point is unstable (in both directions), which means that the weights tend to drift away from it (figure 4; see Text S1).

We now examine the influence of the outlying, unstable fixed point on the dynamics within the allowed region of synaptic values when the baseline firing rates of the two neurons are equal. If the initial weights are fairly close to each other (figure 4A, red area), they eventually end up at the attractor in the upper-right corner of the phase space due to repulsion from the outlying, unstable fixed point. This attractor corresponds to strong recurrent connections. Trajectories of weights that hit the upper boundary (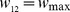) perpendicularly, form another fixed point that is unstable (figure 4, open circle on top). Trajectories to the left of this critical line are eventually absorbed by the attractor at the top-left corner (corresponding to a unidirectional connection), while trajectories to its right are absorbed by the top-right attractor (corresponding to recurrent connections). A similar unstable fixed point exists on the rightmost boundary (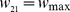; figure 4, open circle on the right). As a result, the state-space of the weights is partitioned into three basins of attraction: one leading to the attractor corresponding to recurrent connections (figure 4, red shading) and the others to attractors that produce unidirectional connections (yellow and green shadings).

**Figure 5:**
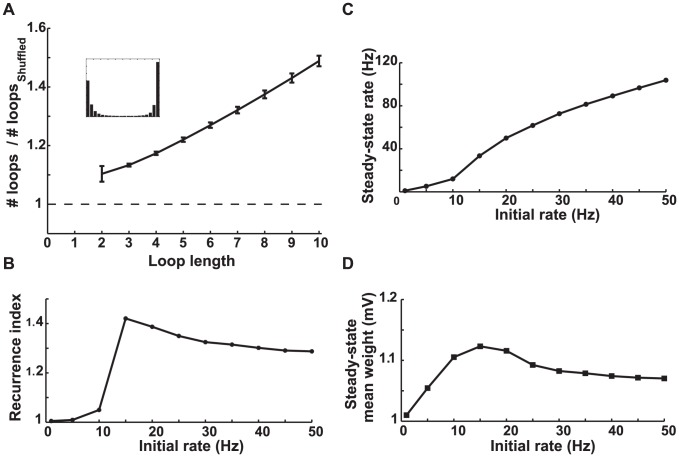
Simulation results for a network with potentiation-dominated STDP. **A.** The number of loops in the steady-state weight matrix divided by the number of loops in a shuffled version of this matrix, as a function of the length of the loop. The initial firing rate of the network was 20 Hz. Error bars depict the standard deviations from 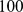 different shuffled versions. The ratios are more than one (dashed line) for all loop lengths, so potentiation-dominated STDP increases the number of loops. The inset shows the final distribution of weights in the network. **B.** Recurrence index of the steady-state weight matrix as a function of the average initial firing rate. The recurrence index is defined as the total number of loops shorter than 10 divided by the same quantity for a shuffled network. The steady-state weight matrix rapidly becomes more recurrent when the initial rate (i.e. the external input) changes from 10 to 15 Hz and then deceases slightly. **C.** The average steady-state firing rate as a function of the average initial firing rate. **D.** The steady-state mean of the synaptic weight as a function of the initial rate.

The appearance of the attractor corresponding to recurrent connections leads to a prediction about networks: STDP with dominant potentiation can generate loops in a network in contrast to balanced STDP. This prediction is confirmed by our numerical simulations showing that there are more loops induced in the steady-state weight matrix of a network in this case (figures 5A).

As the baseline firing rates of the neurons increase, the basin for the attractor with recurrent connections expands (figure 4B, red area). This leads to the prediction that when a network is driven by stronger external input and consequently has a higher initial average firing rate, it will have more loops. Numerical simulation confirms this observation (figure 5B). To quantify the degree of recurrence in a network, we define a “recurrence index” as the sum of the number of loops with less than 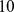 synapses divided by the sum of similar loops in a shuffled version of the network (see Methods). Simulation results show that the recurrence index increases as a function of the initial firing rate of the network and rises rather abruptly when the initial rate exceeds 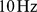, and slightly decreases when the initial rate exceeds 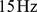 (figure 5B). The eventual decrease of the recurrence index is due to the effect of correlations in the baseline firing, which appear in high rates and are not included in our analysis (see figure S3). The baseline correlations originate from the shared input that the neurons receive from the embedding network and is not related to their pairwise connectivity. Therefore it induces modifications to the reciprocal synapses regardless of the attractor structure explained here.

The existence of the attractor corresponding to the recurrent connections also leads to the prediction that a network modified by potentiation dominant STDP settles into higher steady-state firing rates than a network with balanced STDP, starting from the same initial conditions. The simulations confirm this prediction as well (figure 5C). The steady-state mean synaptic weight is expected to increase as a function of the initial firing rate because the basin of attraction corresponding to recurrent connections expands at high firing rates. The simulation results agree with this expectation up to the initial firing firing rate of 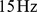, after which the steady-state mean decreases slightly (figure 5D). As in the case of the recurrence index (figure 5B) this decrease is due to the baseline correlations that appear at high firing rates.

### STDP with dominant depression

If depression dominates over potentiation in STDP (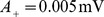 and 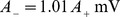 in our examples), the coefficient 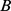 in equations (2) is larger than 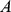 (see Text S1), and the baseline parameter 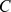 is negative. For these conditions, both elements of the fixed point of the weights, 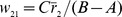 and 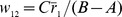, are negative, which is once again outside of the allowed range of synaptic values. In this case, however, the fixed point is a saddle node, which attracts trajectories from one direction and repels them from the other (see Text S1).

**Figure 6:**
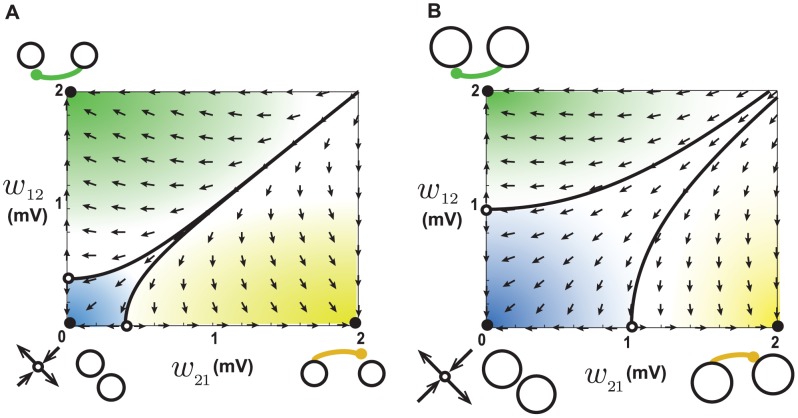
Dynamics of reciprocal synapses when STDP is depression dominated. **A.** When the baseline firing rates of the two neurons are 20 Hz, a saddle node exists out of the allowed range, schematically illustrated at the bottom left. Arrows show the movement of trajectories. Initial conditions starting within the blue area end up at the attractor at the origin, which corresponds to a loss of connectivity. Trajectories that hit the boundaries perpendicularly delineate the borders of the basins of attractions (solid curves). Initial conditions in the yellow area go to the attractor at the bottom right, corresponding to a unidirectional connection from neuron 1 to neuron 2. Initial conditions in the green area go to the attractor at top left, corresponding to a unidirectional connection from neuron 2 to neuron 1. **B.** The same as **A** but for a baseline firing rates of 50 Hz. The basin of attraction for the origin (connectivity loss) becomes larger when the baseline firing rate increases.

As before, we consider two neurons with equal baseline firing rates. The weight trajectories tend to move toward the outlying fixed point in the direction that passes through the origin (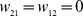; see figure 6A, arrows). This tendency makes the origin an attractor of the dynamics within the allowed range of synaptic weights. This attractor correspond to completely disconnected neurons. Because the outlying fixed point is a saddle node, the trajectories also tend to drift away from it in the direction perpendicular to the positive-slope diagonal. This tendency produces attractors corresponding to unidirectional connections (figure 6, top-left and bottom-right). Once again, trajectories that hit the borders perpendicularly partition the weight space into three basins of attractions corresponding to each attractor (figure 6).

**Figure 7:**
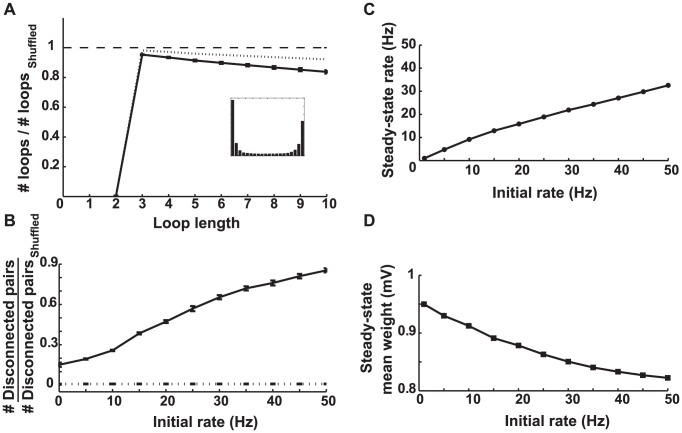
Simulation results of a network with depression-dominated STDP. **A.** The number of loops in the steady-state weight matrix divided by the number of loops in a shuffled version of this matrix, as a function of the length of the loop. The initial firing rate of the network was 20 Hz. Error bars depict the standard deviation from 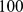 different shuffled versions. The ratios are less than one (dashed line) for all loop lengths. Depression-dominated STDP decrease the loops more efficiently that balanced STDP (figure 3A), which is provided for comparison (dotted line). The inset shows the final distribution of weights in the network. **B.** The number of disconnected pairs in the steady-state weight matrix, divided by the number of disconnected pairs in a shuffled version of the same matrix, as a function of the initial firing rate. The same ratio in the case of balanced STDP is illustrated (dotted line) for comparison. **C.** The average steady-state firing rate as a function of the average initial firing rate. Depression-dominated STDP partially buffers the effect of external input on the average steady-state firing rate. **D.** The steady-state mean synaptic weight as a function of the initial rate.

The dynamics of the synaptic pair we have considered suggests that some pairs of neurons in a network should become disconnected when depression dominates over potentiation. This is a more potent mechanism for eliminating loops than the previous cases, so we expect that STDP with dominant depression eliminates more loops in a large network than the other forms we have considered. Numerical simulations confirm that, indeed, there are fewer loops in the steady-state of a network with depression-dominated STDP compared to the previous cases (compare figure 7A to figures 3A and 5A). In addition, the number of disconnected pairs is large, as predicted (figure 7B).

When the baseline rates of the two neurons increase, the basin of the attractor corresponding to disconnected pair becomes larger (figure 6B). In a newtork, when neurons become excessively active, more connections should thus be eliminated, and the average rate should return to a lower value. Thus, the steady-state firing rate of a network with depression-dominated STDP should be lower than that of a network with balanced STDP starting from the same initial conditions. Simulation results corroborate this observation by showing that the steady-state firing rate of the network increases moderately as a function of the initial firing rate (figure 7C, compare with figure 3E), so depression dominant STDP implements a partial buffering of steady-state firing rates. Finally, the mean synaptic weight is a decreasing function of the initial firing rate in this case (figure 7D).

### STDP with a rightward shifted window

The rightward shifted STDP model, in which nearly synchronous pre- and postsynaptic action potentials induce depression, has been shown to stabilize the distribution of the synaptic weights converging onto a single neuron. The rightward shift can arise from the finite rise time of activation of NMDA receptors [10]. Here, we study this model in the context of a network. The restriction of spike pairings that induce plasticity to those between nearest neighbor pre- and postsynaptic spikes, which is necessary in this case [10], makes the dynamics of the pair of weights more complicated than in the previous cases, because the coefficients 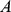, 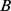 and 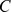 in equations (2) depend on the baseline firing rates (see Text S1). Furthermore, the coefficient 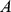 can become negative at high firing rates, which makes the behavior of the system even more complicated. However, if we divide the analysis into three different rate regimes, we can elucidate the full range of behaviors. If depression dominates over potentiation in this model, the synaptic dynamics will be tantamount to the depression-dominant unshifted STDP described above, and the shift only makes depression even more dominant. Novel properties of this model only arise when potentiation dominates over depression, thus we assume that the potentiation domain is larger than the depression domain (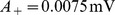 and 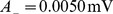 as in [10]), and we set the amount of the shift to be 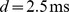.

**Figure 8:**
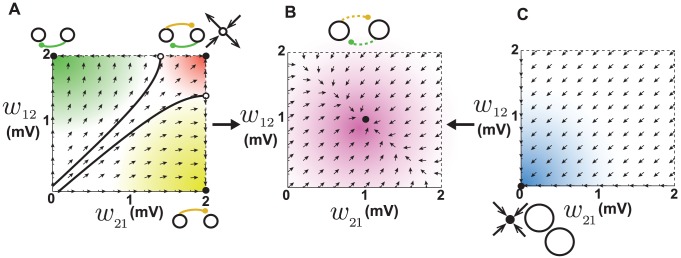
Dynamics of reciprocal synapses with rightward shifted STDP. **A.** When the baseline firing rates of the two neurons are 1.8 Hz, a saddle node exists out of the allowed range, schematically illustrated at the top right. Arrows show the movement of trajectories. Initial conditions starting within the red area end up at the attractor at the top right corner, which corresponds to strong recurrent connections. This increases the baseline firing rate of the embedding network and pushes the network into the regime shown in B. **B.** When the baseline firing rates of the two neurons are 37 Hz, a single stable fixed point exists within the allowed range of synaptic weights. All initial conditions end up at this fixed point, resulting in a recurrent reciprocal connection. **C.** When the baseline firing rates of the two neurons are 50 Hz, a stable fixed point exists out of the allowed range, schematically illustrated at the bottom left. Movement of trajectories toward the stable fixed point results in connectivity loss, regardless of the initial condition. This effect reduces the rate of the embedding network and pushes the system into the regime shown in B. It is not necessary to impose upper bounds in this case, so they are depicted as dotted lines.

When the initial baseline firing rates of the two neurons are low, the coefficients 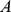, 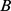 and 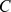 are all positive. This is because the pairing intervals are not typically short enough to fall into the depression domain caused by the shift. In addition, the coefficient 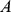 is slightly smaller than 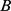. This makes the fixed point for the weights positive and large, meaning that once again it falls out of the putative range of allowed synaptic weights, but this time on the positive not the negative side (figure 8A). We use the term “putative” here because, as we will see, the upper limits on the synaptic weights are not actually required in this case. The fixed point is a saddle node (see Text S1) and attracts the trajectories of weights along the direction toward the top-right corner of the state space (figure 8A, arrows), which corresponds to recurrent connections. This case is qualitatively similar to what we found for STDP with dominant potentiation (compare figures 8A and 4A,B), so the baseline firing rate tends to become higher than its initial value and eventually the dynamics of the system falls into the regime described by figure 8B.

At higher baseline firing rates, the coefficient 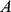 becomes negative. This occurs because the pairing intervals between presynaptic spikes and their causally induced postsynaptic spikes become short enough to fall into the depression domain caused by the shift. This creates a single stable fixed point for the two weights that lies within the putative allowed range of synaptic weights. Both weights are attracted to this fixed point, forming a recurrent connection (figure 8B, arrows; see Text S1), though not of maximal strength.

If the two neurons start with even higher baseline rates, the coefficients 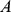 and 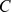 are both negative. This follows because at very high firing rates, even the intervals between randomly paired spikes of the baseline activity are short enough to fall into the depression domain caused by the shift. This pushes the fixed point of the weights out of the allowed range (figure 8C) but, in this case, on the negative side. Because this fixed point is stable, the weights tend to approach it, creating an attractor at the origin that eliminates both weights and disconnects the neurons. This mechanism prunes the weights in the embedding network until the baseline firing rate decreases enough to make the parameter 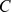 positive. Then, the regime with a stable fixed point within the allowed range (figure 8B) is restored. This is why no upper bounds on the synaptic weights are required in this case.

**Figure 9:**
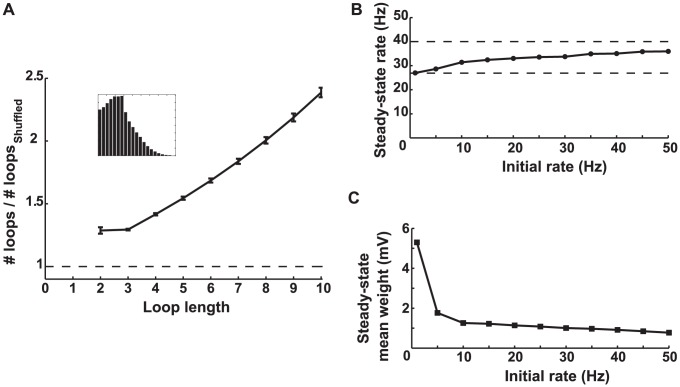
Simulation results for a network with rightward shifted STDP. **A.** The number of loops in the steady-state weight matrix, divided by the number of loops in a shuffled version of this matrix, as a function of the length of the loop. The initial firing rate of the network was 20 Hz. Error bars illustrate the standard deviation from 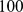 different shuffled versions. The ratios are all greater than one (dashed line), showing that the network generates loops. The inset shows the final distribution of weights in the network. **B.** The average steady-state firing rate as a function of the average initial firing rate. Rightward shifted STDP strongly buffers the effect of the external input on the average steady-state rate, which always ends up in the narrow interval between 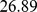 and 40 Hz (dashed lines) predicted by the pair-based analysis (figure S1). **C.** The steady-state mean of the synaptic weight as a function of the initial rate.

Combining these effects, we find that, if the shift is larger than a critical value (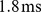 in this case, see figure S1), a network will settle into a regime with a single stable fixed point within a narrow range of steady-state firing rates, regardless of the initial firing rate or the strength of the external input. The condition for this scenario to occur is that the fixed point of the weights becomes stable before it grows negative, as the initial firing rate increases. This happens when the potentiation domain is larger than depression domain and the shift is sufficiently large. The calculations show that a shift of 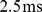 fulfills this condition for our chosen values of potentiation and depression magnitudes (see figure S1). By generalizing from the dynamics of a pair of synapses, two predictions can be made. First, the steady-state matrix of synaptic weights should have many recurrent connections because there is no mechanism to eliminate loops, and reciprocal connections should tend to be strengthened. This prediction is confirmed by numerical simulations that show a highly recurrent steady-state connectivity (figure 9A). Second, because the pairwise connections settle into a regime with a single stable fixed point regardless of the initial baseline rate, the steady-state firing rate of the network should be resilient to changes in the external input or in the initial firing rate. Numerical simulations show that the steady-state firing rate of the network varies very slightly as a function of the initial firing rate (figure 9B). Interestingly, the narrow range of the steady-state firing rates agrees precisely with the prediction of the pairwise analysis (dashed lines in figures 9B and figure S1). Thus, rightward shifted STDP implements a homeostatic mechanism that strongly buffers the steady-state firing rates from external influences. Finally, the mean synaptic weight decreases with increased initial firing rate in this case (figure 9C).

### STDP with a leftward shifted window

A leftward shifted STDP model, in which synchronous pre- and postsynaptic spikes cause potentiation as a result of axonal conduction delays, has been shown to have a desynchronizing effect on population bursts and a synchronizing effect on random spiking in a recurrent network [14]. Here, we study this model within the framework of pairwise analysis. As in the previous section, we consider the interactions of nearest-neighboring spikes. If potentiation dominates over depression in this model, the synaptic dynamics will be tantamount to the potentiation-dominant unshifted STDP described above and the shift only makes potentiation further dominant. Therefore, in order to observe novel behaviors of this model, we assume that the depression domain is larger than potentiation domain (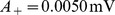 and 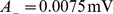), and we set the amount of the shift to be 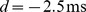, i.e. the parameters are chose to be the flipped versions of those in the rightward shifted model above.

**Figure 10:**
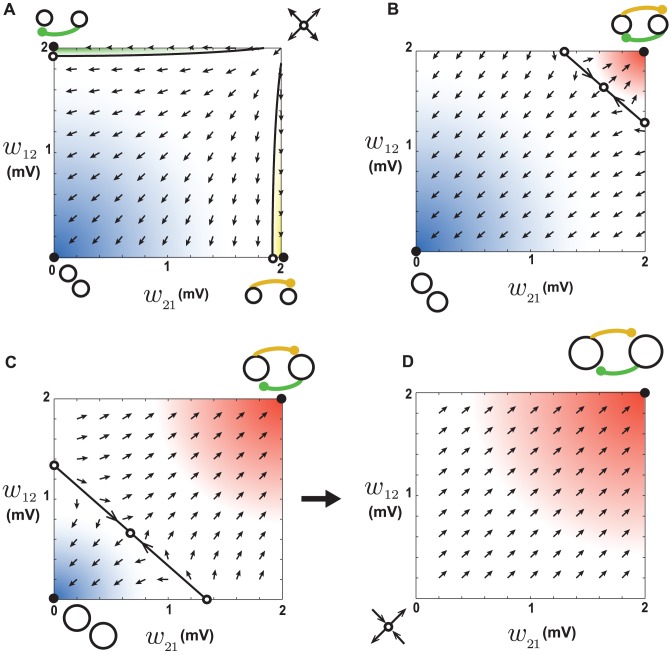
Dynamics of reciprocal synapses with leftward shifted STDP. **A.** When the baseline firing rates of the two neurons are 1.8 Hz, an unstable fixed point exists out of the allowed range, schematically illustrated at the top right. Arrows show the movement of trajectories. Initial conditions starting within the blue area end up at the attractor at the bottom left corner corresponding to connection loss, qualitatively similar to depression dominant STDP (figure 6) **B.** When the baseline firing rates of the two neurons are 35 Hz a saddle node exists within the allowed range of synaptic weights. The initial conditions in the blue area end up at the bottom left attractor (connectivity loss) and the initial condition in the red area end up at the top right (recurrence). **C.** When the baseline firing rates of the two neurons are 38 Hz, the basin of attraction of the recurrent attractor (red) increases. This in turn increases the baseline firing rate and pushes the system into the regime explained in D. **D.** When the baseline firing rates of the two neurons are 50 Hz, a saddle node exists out of the allowed range, schematically illustrated at the bottom left. Movement of trajectories away from this fixed point results in recurrent connections, regardless of the initial condition, so all the weights potentiate up to the maximum allowed value.

When the initial baseline firing rate is low, the coefficients 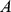 and 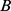 are positive (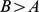) and 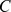 is negative. This is because the pairing intervals are not typically short enough to fall into the potentiation domain caused by the shift. As a result the fixed point is positive, unstable in both directions, and out of the allowed range of weights (figure 10A). The weight trajectories tend to drift away from the fixed point in the direction that passes through the origin, so this behavior is qualitatively similar to what we found for STDP with dominant depression. The attractor at the origin corresponds to completely disconnected neurons, therefore the baseline firing rate tends to become less than its initial value.

For higher initial baseline firing rates, coefficient 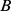 becomes negative, because the pairing intervals between pre- and postsynaptic spikes become short enough to fall into the potentiation domain caused by the shift. This turns the fixed point into a saddle node and pushes it into the allowed range of weights (figure 10B). The weights drift away from the fixed point in the directions that passes through the origin and the top-right corner, and are attracted to it in the perpendicular direction. As a result, both the origin and top-right corner turn into attractors, corresponding to disconnected and recurrently connected neurons respectively (figure 10B, closed circles). Because these two points are the only attractors of the system, the network is expected to become highly recurrent in this case and the neurons to become either recurrently connected or disconnected. This regime happens for a narrow range of initial firing rates. As the initial firing rate increases, the basin of the top-right attractor becomes larger (figure 10C). As a result, more recurrent connections form and hence the baseline firing rate increases, which eventually pushes the system into the regime described in the following paragraph.

For even higher initial baseline firing rates, not only coefficient 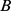 becomes negative, but also 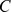 turns positive and the fixed point is pushed out of the allowed range on the negative side (figure 10D). It remains a saddle node, so the weights are repelled from it in the direction that passes through the top-right corner, which becomes the only attractor of the system corresponding to recurrent connection. Therefore, it is expected that all the synapse in the network potentiate up to the upper limit of the weights in this case.

**Figure 11:**
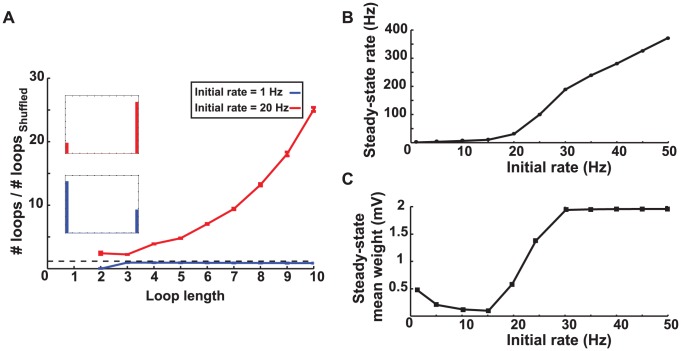
Simulation results for a network with leftward shifted STDP. **A.** The number of loops in the steady-state weight matrix, divided by the number of loops in a shuffled version of this matrix, as a function of the length of the loop. The initial firing rate of the network was 5 Hz for the blue curve and 25 Hz for the red curve. Error bars illustrate the standard deviation from 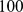 different shuffled versions. The ratios are smaller than one (dashed line) for the blue curve, showing that leftward shifted STDP eliminates loops at low firing rates. The ratios are all greater than one (dashed line) for the red curve, and higher than all other cases (compare with figures 5A and 9A). Thus, leftward shifted STDP is a very potent loop generator at higher rates. The inset shows the final distribution of weights in the network in these two cases (colors matched). **B.** The average steady-state firing rate as a function of the average initial firing rate. **C.** The steady-state mean synaptic weight as a function of the initial rate. For low initial rates, it is a decreasing function, for intermediate initial rates it increases, and for high initial rates in hits the upper boundary.

In summary, the above description shows that as the initial baseline firing rate increases, the networks undergoes three different phase: 1) for low initial rates it behaves similarly to depression-dominant STDP, i.e. recurrent connections are eliminated and the steady-state firing rate is partially buffered; 2) for higher initial rates the network becomes highly recurrent and the steady-state rate increases; 3) for even higher initial rates, all the weights become potentiated up to the maximum, and the firing rate is pathologically high. The simulation results confirm these predictions. When the initial rate is less than 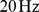, the steady-state rate increases modestly (figure 11B, left) and the the mean of synaptic weights decreases (figure 11C, left) as a function of initial rate. The number of loops also decrease in this regime (figure 11A, blue). For higher initial rates, the mean synaptic weight and the steady-state rate increase rapidly (figures 11B-C, middle) and the network is highly recurrent (figure 11A, red). For initial rates higher than 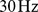, the mean synaptic weight equals the maximum allowed value, implying that all the weights are maximally potentiated (figure 11C, right), and the steady-state rate is pathologically high. Although the simulation results qualitatively show the full range of behaviors predicted by pairwise analysis, the initial firing rate at which the transitions occur in simulations is lower than that predicted from calculations (see Text S1). This discrepancy is due to baseline correlations that appear at high rates (see figure S3). In presence of baseline correlations, the neurons tend to fire synchronously regardless of their pairwise connections, and hence the synapses get potentiated indiscriminately due to leftward shift of the STDP.

## Discussion

By analyzing pairwise interactions of neurons affected by STDP, we clarified how conventional pair-based STDP functions as a loop-eliminating mechanism in a network of spiking neurons and organizes neurons into in- and out-hubs, as reported in [21]. Loop-elimination increases when depression dominates, and turns to loop generation when potentiation dominates. STDP with dominant depression implements a partial buffering mechanism for network firing rates. Rightward shifted STDP can generate recurrent connections in a network and functions as a strict buffering mechanism to maintain a roughly constant network firing rate. STDP with leftward shift functions as a partial buffer of firing rates and a loop eliminator at low rates, and as a potent loop generator at higher rates.

All of our analytical results were obtained by considering the effect of imposing weight constraints on a linear system describing pairwise interactions of neurons in the presence of STDP. The effect of constraints on Hebbian plasticity has been explored before to explain the formation of visual receptive fields [26]. Our work can be viewed as an extension of this approach to a specific form of Hebbian plasticity that involves the timing of spikes, namely STDP. In the context of a recurrent network, this method can predict the outcome of STDP in shaping the connectivity of the network and qualitatively captures the direction of change of firing rates in the network. However, the steady-state firing rate of the network cannot be quantitatively calculated by this approach, since the analysis is focused on the snapshots of the weight dynamics given the current firing rates.

The network used in our numerical simulations was densely connected so that every neuron could potentially form a synaptic connection to every other one. However, our analytical results does not rely on any particular assumption about the density or sparsity of network connectivity. Instead, the results indicate that STDP can organize patterns of connectivity in particular ways within the framework provided by anatomical constraints, developmental hard-wiring and other physiological mechanisms, such as other forms of plasticity.

In a series of articles, Gilson and colleagues studied the structures that arise from STDP in a recurrent network in response to the patterns of correlations in the external input [15]–[20]. Here, we took a different approach. We focused on the network structures that arise in the absence of correlations either imposed by external input or originated from common inputs within the network, inspired by the observation that these are dramatically reduced by fast and strong recurrent inhibition [25]. Instead, we systematically studied the effect of the shape of the STDP window on the structures that arise in this decorrelated state. Our results can be viewed as a basis over which any structures induced by external correlations will be mounted.

A prominent feature of STDP is its ability to organize neurons into in- and out-hubs. The dependence of hub-formation on baseline firing rate shows how heterogeneity at the level of external inputs can influence the internal structure of a neural network. Moreover, this property of STDP can play an important protective role in pathological cases in which a sub-population of excitatory neurons fires at atypically high rates. Through STDP, most of the incoming synapses to this sub-population will be weakened to mitigate the excessive high firing rate. Decoupling of a highly active sub-population from an embedding network through STDP has been observed previously in networks with an excitation-inhibition balance [13].

A related study based on simulations of a small network showed that depending on the external input, an STDP rule that is phenomenologically similar to the triplet model [27] can either induce feedforward structures or recurrent connections, which was argued to be incompatible with simple pair-based STDP [22]. Although our study only addressed structures arising from pair-based STDP, our results show that recurrent connections can arise if potentiation dominates depression or the plasticity window is shifted. Interestingly, the dependence of the structures on external input in the case of leftward shifted STDP is similar to that of the more elaborate model studied by Clopath and colleagues [22].

A number of studies indicate that, apart from the timing of spikes, several other factors including firing rates, inhibitory inputs, dendritic spikes and neuromodulation influence plasticity induction [27]–[30]. Various STDP rules (including the multi-spike STDP models reviewed in [31]) have been proposed to incorporate some of these factors. The method we have developed can be used with these other STDP models, but we did not include an analysis of multi-spike STDP or more complex models because we did not want an excessive number of examples nor complexity in the STDP rule to obscure the basic approach and the insights that it provides.

The ability of pair-based STDP to generate recurrent connections has been shown previously [11]. Although in that case the depression domain was elongated, but the magnitude of potentiation domain was larger such that overall potentiation was dominant over depression, which agrees with our results on loop generation through STDP. Lubenov and colleagues have shown that STDP with leftward shifted window, arising from axonal conduction delays, can generate recurrent connections and thereby synchronize neurons when the network is initialized with a tonic irregular firing mode. In the bursting mode, leftward shifted STDP has the opposite effect, i.e. it eliminates loops and desynchronizes the neurons [14]. Because the networks we studied were in excitatory/inhibitory balanced state in which the firing patterns are irregular and asynchronous [28]–[30], our findings about loop generation through leftward shifted STDP agree with the results of [14], even though the same model can function as a loop eliminating mechanism at low initial firing rates.

A combination of axonal, synaptic and dendritic propagation delays can induce a leftward shift in STDP window [14]. On the other hand, the finite rise time of the NMDA receptor activation can give rise to a rightward shift in the window [10]. Thus the exact magnitude and direction of the shift depends on the relative contribution of these opposing factors. For instance, because the back-propagating postsynaptic spikes arrive at distal synapses with a longer delay than at proximal ones, leftward shifted STDP is expected to be observed more in the distal dendrites and rightward shift is expected at proximal sites. Moreover, the relative magnitude of potentiation and depression varies considerably along the dendritic tree [31]–[34]. Therefore, each of the different versions of STDP window analyzed in our study may be relevant in a particular region of the dendritic tree. A general prediction of our study is then that different regions of the dendritic tree may participate in different network structures as a result of differences in their STDP windows.

In a number of studies, clusters of three or four synaptically connected neurons have been observed in cortical slices at a higher frequency than expected from a random or distance-based connectivity pattern [35], [36]. We doubt that STDP can account for these clusters unless network synapses were unrealistically strong, so strong that the causal effect of single spikes from one neuron can pass through two or more synapses and transiently increase the firing rate of another neuron. Otherwise the effects of STDP would be restricted to mono-synaptically connected neurons, even in larger ensembles. In fact, our results show that loops of length 3 are usually the loops least affected by STDP. This can be explained by the direct effect of STDP being confined to loops of length 2. In loops of length 3, unlike longer loops, there is no contribution from reciprocally connected pairs of neurons (loops of length 2).

In conclusion, studying pairwise interactions of neurons through STDP provides a number of important insights about the structures that arise from this plasticity in large networks. This approach can be extended to networks with more complex STDP models and more structured external input.

## Methods

### The Neuron model

We used leaky integrate-and-fire (LIF) model neuron in our simulations. The membrane potential of the LIF neuron obeys
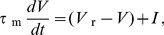
(3)where 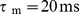 is the membrane time constant, 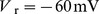 is the resting potential, and 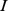 is the synaptic input (see below). Although the input appears as a current, it is actually measured in units of the membrane potential (mV) because a factor of the membrane resistance has been absorbed into its definition. When the membrane potential 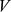 reaches the firing threshold 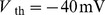 , the neuron fires an action potential and the membrane potential resets to the resting value 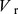.

### Network model

A network of 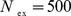 excitatory and 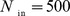 inhibitory LIF neurons was simulated. Each neuron receives excitatory and inhibitory inputs from all the other neurons in the network. The strengths of the excitatory-to-inhibitory, inhibitory-to-excitatory and inhibitory-to-inhibitory synapses are fixed. At the beginning of each simulation, their strengths were drawn from uniform distributions ranging from 0 to 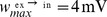, 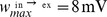, and 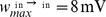 respectively. The excitatory-to-excitatory connections are modified by pair-based STDP as described below. They are also initialized at the beginning of each run to random values from a uniform distribution ranging between 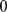 and 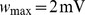. Although the inhibitory connections are stronger than excitatory connections (but inhibitory-to-excitatory and inhibitory-to-inhibitory connections are equally strong), the network settles into an excitation/inhibition balanced state with these initial conditions (see figure S3). In this state, individual neurons fire irregularly and asynchronously [28]–[30] and the strong recurrent inhibition causes the firing correlations due to shared input to be very week [25]. The connections are all to all and self connections are prohibited for all neurons.

Each presynaptic action potential arriving at an excitatory or inhibitory synapse induces an instantaneous jump or fall in synaptic input respectively, by an amount proportional to the appropriate synaptic weight. The input decays exponentially between presynaptic action potentials. In addition to synaptic inputs originating from the neurons within the network, the input to each neuron includes an external constant bias term and independent white noise. Taken together, the input to the 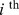 excitatory or inhibitory neuron is described by
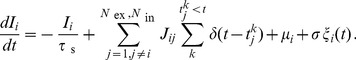
(4)Here, the synaptic time constant is 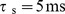, 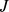 denotes the full matrix of connections (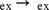, 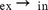, 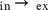 and 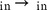) and the first sum runs over all neurons (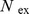 and 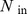 for excitatory and inhibitory populations, respectively). The second sum runs over all the times 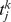 of spikes produced by neuron 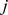 prior to time 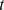, indexed by 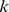. The parameters 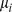 and 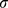 determine the mean and the variability of the input (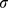 has not subscript 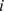 because it is the same for all neurons), and 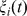 satisfies 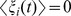 and 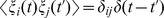, with the brackets denoting averages. The parameter 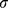 was set to 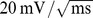 to provide an average initial baseline firing rate of 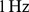 for the neurons in the network when 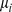 is zero. In the simulations, the value of 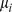 was changed systematically to modify initial firing rates. Each simulation is run until the excitatory-to-excitatory connections reached a steady-state in which the average firing rate, and the mean and variance of the synaptic weights remained constant.

### Counting loops

To count the number of closed loops implied by the matrix of excitatory-to-excitatory synaptic weights (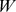), we first turn the network into a directed graph [21]. This is done by comparing each synaptic weight to a threshold value 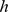, and assigning the value 1 to the synapse if its weight is greater than or equal to 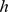, and assigning a zero otherwise. This defines the adjacency matrix 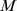 of the resultant directed graph, which can be written formally as
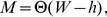
(5)where 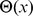 is the Heaviside step function. The number of closed loops of length 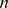 in the adjacency matrix 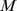 is then
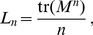
(6)where 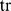 denotes the matrix trace (the sum of the diagonal elements). To evaluate the degree of recurrence in a network, we compare the number of closed loops obtained from the above equation with the number in a randomly permuted (shuffled) version of the same matrix. This distinguishes recurrent connections formed by chance from those that arise from plasticity. In the following sections, whenever we mention the number of loops in a network, we are in fact referring to the number of loops in the adjacency matrix formed by turning the network into a directed graph as described above. In addition, when we refer to a “number” of synapses, we really refer to the number of synapses with strengths greater than the threshold 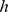. To obtain a loop count that is not biased by the overall strengths of the weights, we chose 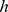 to be equal to the mean of the excitatory synaptic weights. For figures 3, 5, 7, 9, 11 (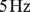 initial rate) and 11(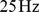 initial rate) , respectively, 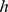 was set to 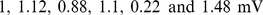.

## Supporting Information

Figure S1
**Parameter range for stability in rightward shifted STDP.** The gray area shows the range of baseline firing rates at which a stable positive fixed point for reciprocal synapses exists, as a function of the shift (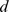). Other parameters of the STDP window are 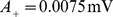, 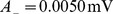 and 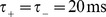. For 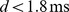 no positive stable fixed point exists. For 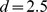 (dotted line) which was used in simulations, the upper and lower bounds of the baseline rates supporting a stable positive fixed point are 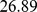 and 40 Hz respectively (dashed lines). The steady-state firing rate is predicted to end up in this range, which agrees with simulations (figure 9B).(EPS)

Figure S2
**Dynamics of reciprocal synapses with weight-dependent STDP (soft bounds).** The nulclines 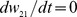 and 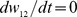 are depicted by yellow and green curves, respectively. The top row shows the results for balanced STDP (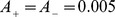), the middle row shows the results for potentiation-dominant STDP (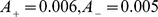), and the bottom row shows the results for depression-dominant STDP (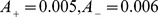). The columns correspond to different baseline firing rates. The position of the fixed point changes very slightly by changing the parameters. In all of the panels 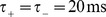.(EPS)

Figure S3
**The average cross-covariance of spike trains of excitatory neurons in the simulated networks.** The first row shows the results for the initial state of the network, when STDP is not yet started. Next rows show the results for the final network (steady-state) for each STDP model. The columns correspond to different initial firing rates, induced by external input. The inset number in each panel is the average coefficient of variation (CV) of inter-spike-intervals. The gray panels show the cases where the average cross-covariance significantly deviate from zero and the CV is much smaller than 1, i.e. the uncorrelated asynchronous irregular state becomes disrupted. In all panels, the cross-covariance and the CV are averaged over 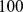 randomly chosen neurons (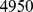 pairs for cross-covariance), the duration of spike trains is 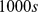, and the bin size is 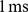.(EPS)

Text S1 **Supplementary information.** This file includes the details of deriving the equations for pairwise interactions of weights, shifted STDP, STDP with soft bounds, and the calculation of cross-covariance of spike trains in the network.
(PDF)
