# Supplementary material for: Pairwise Analysis Can Account for Network Structures Arising from Spike-Timing Dependent Plasticity
Source: PLoS Comput Biol. 2013 Feb 21;9(2):e1002906. doi: 10.1371/journal.pcbi.1002906 (PMC3578766; doi:10.1371/journal.pcbi.1002906)
Supplement: Text S1 — Supplementary information. This file includes the details of deriving the equations for pairwise interactions of weights, shifted STDP, STDP with soft bounds, and the calculation of cross-covariance of spike trains in the network. (PDF) [file pcbi.1002906.s004.pdf]

Supplementary Information for: Pairwise Analysis Can Account for Network Structures Arising from Spike-Timing Dependent Plasticity

Baktash Babadi, L. F. Abbott

Deriving the equations for pairwise interactions of weights

Synaptic weights are modified through STDP as a function of the interval between pre- and postsynaptic spikes (Δt) according to equation(1). Assuming an all-to-all interaction and Poisson statistics for the spike trains, the probability distribution of pairing intervals with a given interval between the pre- and postsynaptic spike times is simply the product of their rates. The rate of each neuron has a baseline value ($\bar{r}_1$ and $\bar{r}_2$, respectively), and it is transiently increases at a times t after the arrival of a spike from the other neuron, due to the presence of the excitatory synapse, so that

$$r_1(t) \approx \bar{r}_1 + \frac{w_{12} \exp\big(-t/\tau_{\rm s}\big)}{(V_{\rm th} - V_{\rm r})\tau_{\rm m}}, \quad \text{and} \quad r_2(t) \approx \bar{r}_2 + \frac{w_{21} \exp\big(-t/\tau_{\rm s}\big)}{(V_{\rm th} - V_{\rm r})\tau_{\rm m}},$$

where $\tau_{\rm s}$ is the synaptic time constant, $\tau_{\rm m}$ is the membrane time constant, $V_{\rm th}$ is the firing threshold, $V_{\rm r}$ is the resting potential, and w_{12} and w_{21} are the synaptic weights from neuron 1 to neuron 2 and vice versa. For w_{21} the transient increase of the rate of neuron 2 falls into the potentiation domain of STDP, and the transient increase of the rate of neuron 1 falls into the depression domain. Therefore, the average drift of w_{21} is

$$\begin{aligned} \frac{dw_{21}}{dt} &= A_+ \int_0^{+\infty} dt\, \bar{r}_1\, r_2(t)\, \exp(-t/\tau_+) - A_- \int_{-\infty}^{0} dt\, \bar{r}_2\, r_1(t)\, \exp(t/\tau_-) \\ &= A\,\bar{r}_1\, w_{21} - B\,\bar{r}_2\, w_{12} + C\,\bar{r}_1\,\bar{r}_2 \end{aligned} \tag{S1}$$

with coefficients

$$\begin{aligned} A &= \frac{A_+\,\tau_+\,\tau_{\rm s}}{\tau_{\rm m}\,(V_{\rm th} - V_{\rm r})\,(\tau_+ + \tau_{\rm s})} \\ B &= \frac{A_-\,\tau_-\,\tau_{\rm s}}{\tau_{\rm m}\,(V_{\rm th} - V_{\rm r})\,(\tau_- + \tau_{\rm s})} \\ C &= A_+\,\tau_+ - A_-\,\tau_- \,. \end{aligned} \tag{S2}$$

In the above equations A_+ and A_- specify the maximum potentiation and depression, and τ_+ and τ_- determine the temporal spread of the STDP window for potentiation and depression. The system of equations (2) are obtained by combing these results with a similar calculation for w_{12}.

Dynamics of the pair of weights

Equations (2) can be expressed in vector form as

$$\begin{bmatrix} \dot{w}_{21} \\ \dot{w}_{12} \end{bmatrix} = \begin{bmatrix} A\,\bar{r}_1 & -B\,\bar{r}_2 \\ -B\,\bar{r}_1 & A\,\bar{r}_2 \end{bmatrix} \begin{bmatrix} w_{21} \\ w_{12} \end{bmatrix} + C\,\bar{r}_1\,\bar{r}_2\,. \tag{S3}$$

The eigenvalues of the above matrix of coefficients, which govern the dynamics of the system, are

$$\lambda_\pm = \frac{A\,(\bar{r}_1 + \bar{r}_2) \pm \sqrt{A^2\,(\bar{r}_1 - \bar{r}_2)^2 + 4B^2\,\bar{r}_1\,\bar{r}_2}}{2} \tag{S4}$$

with the associated eigenvectors $(1, (A\,r_1 - \lambda_\pm)/(B\,r_2))$. When $\bar{r}_1 = \bar{r}_2$, these eigenvectors are simply $(1, \pm 1)$.

In the case of balanced STDP ($A = B$), one eigenvalue is zero and the other is positive. Therefore, the weights drift away from the equilibrium line. In the case of potentiation-dominated STDP ($A > B$), both eigenvalues are positive, so the fixed point is unstable. In the case of depression-dominated STDP ($A < B$), one eigenvalue is positive and the other negative, so the fixed point is a saddle node.

Assuming that the baseline rates are equal ($\bar{r}_1 = \bar{r}_2 = \bar{r}$), for the case of potentiation-dominated STDP, the point where w_{21} hits its maximum and $dw_{12}/dt = 0$ (i.e. the end point of the trajectory perpendicular to the rightmost boundary, figure 4) is

$$w_{21} = w_{\max}\,,\; w_{12} = \frac{B\,w_{\max} - C\,\bar{r}}{A}\,. \tag{S5}$$

This point defines the boundary between the basins of attraction (figure 4). As equation S5 shows, w_{12} becomes smaller when the baseline rate increases, and the boundary moves downward. Consequently, the basin of attraction for the top-right corner (figure 4) grows.

In the case of depression-dominated STDP, the point at which w_{21} hits zero and $dw_{12}/dt = 0$ (i.e. the trajectory perpendicular to the rightmost boundary) is

$$w_{21} = 0\,,\; w_{12} = \frac{-C\,\bar{r}}{A}\,. \tag{S6}$$

This point defines the boundary between the basins of attraction (figure 6). C is negative in this case, thus as the baseline rate increases, w_{12} becomes larger, the boundary moves upward, and the basin of the attractor at origin (figure 6) grows. Similar arguments apply for the other boundaries in figures 4 and 6.

The drift of the pair of synapses under shifted STDP

In the shifted STDP model, only nearest-neighboring pre- and postsynaptic spike pairs participate in plasticity. We assume that the spike trains of neurons 1 and 2 are Poisson. In this case, the probability density that neuron 2 fires an interval t after a spike from neuron 1, and no other spike occurs between these two (i.e. nearest-neighbor condition), is

$$P_{1\to 2}(t) = \bar{r}_1\, r_2(t)\, \exp\Big[- \int_0^t ds\, \big(\bar{r}_1 + r_2(s)\big)\Big]\,. \tag{S7}$$

Because the spike of neuron 1 precedes that of neuron 2, the firing rate of neuron 2 increases transiently during the pairing interval (equation S1). Assuming that $w_{21} \ll V_{\text{th}} - V_r$, the above probability can be approximated as

$$\begin{aligned} P_{1\to 2}(t) \;\; &\approx \;\; \bar{r}_1\, \bar{r}_2\, \exp\Big[- (\bar{r}_1 + \bar{r}_2)\, t\Big] \\ &\quad + w_{21}\, \frac{\bar{r}_1\, \tau_{\text{s}}}{\tau_{\text{m}}\, (V_{\text{th}} - V_{\text{r}})}\, \exp(-\bar{r}_1\, t)\, \Big[\gamma_2\, \exp(-\lambda_2 t) - \bar{r}_2\, \exp(-\bar{r}_2 t)\Big]\,, \end{aligned} \tag{S8}$$

where $\gamma_2 = \bar{r}_2 + 1/\tau_s$. The drift of w_{21} for rightward shift ($d > 0$) can now be calculated as

$$
\begin{aligned}
\frac{dw_{21}}{dt} &= A_+ \int_d^{+\infty} dt\, P_{1\to 2}(t)\, \exp\left[-(t-d)/\tau_+\right] \\
&\quad -A_- \int_0^{d} dt\, P_{1\to 2}(t)\, \exp\left[(t-d)/\tau_-\right] \\
&\quad -A_- \int_0^{+\infty} dt\, P_{2\to 1}(t)\, \exp\left[-(t+d)/\tau_-\right] \\
&= A(\bar{r}_1,\bar{r}_2)\,\bar{r}_1\, w_{21} - B(\bar{r}_1,\bar{r}_2)\,\bar{r}_2\, w_{12} + C(\bar{r}_1,\bar{r}_2)\,\bar{r}_1\,\bar{r}_2
\end{aligned}
\tag{S9}
$$

with coefficients

$$
\begin{aligned}
A(\bar{r}_1,\bar{r}_2) &= \frac{1}{\tau_{\mathrm{m}}\,(V_{\mathrm{th}} - V_{\mathrm{r}})} \left(\frac{A_+\,\tau_{\mathrm{s}}\,(\tau_+ + d)\,(1+\bar{r}_1\tau_+)}{(1+\hat{r}\,\tau_+)\,(\tau_{\mathrm{s}} + \tau_+ + \hat{r}\,\tau_{\mathrm{s}}\,\tau_+)} - (A_+ + A_-)\,d \right) \\
B(\bar{r}_1,\bar{r}_2) &= \frac{A_-\,\tau_{\mathrm{s}}\,(\tau_- - d)\,(1+\bar{r}_2\tau_-)}{\tau_{\mathrm{m}}\,(V_{\mathrm{th}} - V_{\mathrm{r}})\,(1+\hat{r}\,\tau_-)\,(\tau_{\mathrm{s}} + \tau_- + \hat{r}\,\tau_{\mathrm{s}}\,\tau_-)} \\
C(\bar{r}_1,\bar{r}_2) &= \frac{A_+\,\tau_+\,(1-\hat{r}\,d)}{1+\hat{r}\,\tau_+} - \frac{A_-\,\tau_-\,(1+\hat{r}\,d)}{1+\hat{r}\,\tau_-}\,,
\end{aligned}
\tag{S10}
$$

where $\hat{r} = \bar{r}_1 + \bar{r}_2$. Note that, in this case, the coefficients are functions of the baseline rates.

The case $d = 0$ is equivalent to unshifted STDP with nearest-neighbor interactions. The above coefficients show that in this case the model has the same qualitative behavior as STDP with all-to-all spike interactions, because the rate-dependence does not change the fixed points and eigenvalues qualitatively.

For $d > 0$ (rightward shifted window), the coefficient A decreases and eventually becomes negative as the baseline rate increases. This is because the first term in the parenthesis, which is positive, decreases with rate, while the second term, which subtracts a positive amount, remains constant. At high firing rates, the coefficient C also becomes negative, because its first term (positive) decreases with rate while the second term increases. Assuming that the baseline firing rates of the two neurons are the same ($\bar{r}_1 = \bar{r}_2 = \bar{r}$), the eigenvalues of the system are $\lambda_\pm = (A \pm B)\,\bar{r}$, with associated eigenvectors $(1, \mp 1)$. For sufficiently negative A both eigenvalues become negative and the fixed point becomes stable. The location of the fixed point ($w^* = C\bar{r}/(B - A)$) depends on the sign of the coefficient C. In order to have a positive stable fixed point, a range of baseline rates should exist in which both eigenvalues are negative but the coefficient C is still positive. As argued in the results section, if such a fixed point exists the network settles into it regardless of the strength of the external input and the initial conditions. Also since the fixed point is stable, it is not necessary to impose an upper bound of the synaptic weights in this case; all the weights will be attracted to the fixed point even if they start from a higher value. The critical baseline firing rate at which λ_+ becomes negative can be calculated numerically from equations (S10) (figure S1, lower boundary of gray area). Note that when λ_+ is negative, λ_- is also necessarily negative. Equations (S10) also shows that the coefficient C becomes negative when $\bar{r} > (A_+ - A_-)/(2(A_+ + A_-)d)$ (figure S1, upper boundary of gray area) provided that $\tau_+ = \tau_-$. This also shows that for C to change sign as a function of the baseline rate, A_+ should be larger than A_-, which motivated our choice of parameters in the results section. As figure S1 shows (gray area), when the shift (d) is larger than a critical value there is a range of baseline rates for which both eigenvalues are negative while C (and hence the fixed point) is positive. For our choice of window parameters in the results section, the critical shift is around 1.8 ms. We used a shift of 2.5 ms in our simulations. The above calculations also provide

the exact range of baseline rates in which the stable positive fixed point exists (figure S1, dashed line). The steady-state rate of the network is predicted to lie in this range, as confirmed by simulations (figure 9).

The coefficients in the case of leftward shifted STDP ($d < 0$), calculated in the same way as in the rightward shifted case above, are:

$$
\begin{aligned}
A(\bar{r}_1, \bar{r}_2) &= \frac{A_+ \, \tau_\mathrm{s} \, (\tau_+ + d) \, (1 + \bar{r}_2 \tau_+)}{\tau_\mathrm{m} \, (V_\mathrm{th} - V_\mathrm{r}) \, (1 + \hat{r} \, \tau_+) \, (\tau_\mathrm{s} + \tau_+ + \hat{r} \, \tau_\mathrm{s} \, \tau_+)} \\
B(\bar{r}_1, \bar{r}_2) &= \frac{1}{\tau_\mathrm{m} \, (V_\mathrm{th} - V_\mathrm{r})} \left(\frac{A_- \, \tau_\mathrm{s} \, (\tau_- - d) \, (1 + \bar{r}_1 \tau_-)}{(1 + \hat{r} \, \tau_-) \, (\tau_\mathrm{s} + \tau_- + \hat{r} \, \tau_\mathrm{s} \, \tau_-)} + (A_+ + A_-) \, d \right) \\
C(\bar{r}_1, \bar{r}_2) &= \frac{A_+ \, \tau_+ \, (1 - \hat{r} \, d)}{1 + \hat{r} \, \tau_+} - \frac{A_- \, \tau_- \, (1 + \hat{r} \, d)}{1 + \hat{r} \, \tau_-} \, ,
\end{aligned}
\tag{S11}
$$

where $\hat{r} = \bar{r}_1 + \bar{r}_2$. As the baseline firing rate increases, the coefficient B decreases and eventually becomes negative, and the coefficient C increases and becomes positive. To observe the full range of behaviors described in the results section, there should be range of baseline firing rates at which the fixed point is a positive saddle node, i.e. $\lambda_+ < 0$ and $C < 0$. Due to the symmetry between the coefficients in the rightward and leftward shifted models (equations (S10) and (S11)), the same parameters chosen for rightward shifted STDP fulfill these conditions provided that potentiation parameters are interchanged with depression parameters and the sign of the shift is flipped ($A_+ \to A_-$, $A_- \to A_+$, $d \to -d$), which is what we did in the results section. Because the fixed point never becomes stable in this case, it is necessary to impose lower and upper bounds. Therefore, an important critical baseline rate in this case is when the fixed point enters the allowed range of synaptic weights. Given our chosen parameters, this critical rate is calculated to be 33.8 Hz. However, this transition happens at a lower rate in numerical simulations. This discrepancy is due to the baseline correlations that tend to be strong in the case of leftward shifted STDP, as this plasticity rule strongly enhances recurrent connections and hence increases correlations due to shared input. Moreover, this version of plasticity is highly sensitive to these correlations because nearly synchronous pre- and postsynaptic spikes induce potentiation regardless of their ordering.

Pairwise interactions in STDP with soft bounds

Instead of imposing hard bounds on the minimum and maximum synaptic weights, the ranges of synaptic strengths can be confined by imposing soft bounds, i.e. making the potentiation and depression weight-dependent (see ref [24]):

$$
\Delta w = \begin{cases} -\dfrac{w}{w_{max}} A_- \, e^{\Delta t/\tau_-} & \text{if } \Delta t \le 0 \\[2ex] \left(1 - \dfrac{w}{w_{max}}\right) A_+ \, e^{-\Delta t/\tau_+} & \text{if } \Delta t > 0 \, , \end{cases}
\tag{S12}
$$

where w_{max} is the maximum allowed synaptic weight. Without loss of generality, we assume that $w_{max} = 1$. Using the same method introduced above, the pairwise dynamics of reciprocal synapses through weight-dependent STDP is governed by:

$$
\begin{aligned}
\frac{dw_{21}}{dt} &= A \, \bar{r}_1 \, w_{21} \, (1 - w_{21}) - B \, \bar{r}_2 \, w_{12} \, w_{21} + (C - 2D) \, \bar{r}_1 \, \bar{r}_2 \, w_{21} + D \, \bar{r}_1 \, \bar{r}_2 \\
\frac{dw_{12}}{dt} &= A \, \bar{r}_2 \, w_{12} \, (1 - w_{12}) - B \, \bar{r}_1 \, w_{21} \, w_{12} + (C - 2D) \, \bar{r}_1 \, \bar{r}_2 \, w_{12} + D \, \bar{r}_1 \, \bar{r}_2 \, .
\end{aligned}
\tag{S13}
$$

Here, the coefficients A, B and C are the same as in equation (S2), and $D = A_+ \tau_+$. Unlike the case of STDP with hard bounds, equations (S13) are nonlinear. The fixed points of this system are the intersections of nullclines of the two equations, i.e. the curves corresponding to $dw_{21}/dt = 0$ and $dw_{12}/dt = 0$, respectively (figure S2). As can be seen, the system has only one stable fixed point in the middle of the allowed range, which varies very slightly by changing the baseline firing rates or the potentiation/depression balance of STDP. Therefore, we conclude that the synapses do not explore the whole range of their allowed strengths when soft bounds are imposed. This is the motivation for us to focus our analysis to the STDP with hard boundaries in the article.

Cross-covariance of spike trains in the network

One of the assumption of our analysis for pairwise dynamics of synapses is that the baseline firing rates of neurons are uncorrelated. The correlations between the spike trains of the neurons in the network are expected to be insignificant because of the fast and strong recurrent inhibition (see ref [25]). In order to check whether this is the case, we calculated the cross-covariance between the spike trains of pairs of excitatory neurons in our simulated networks, both in their initial condition and after stabilization of synaptic weights which are modified through STDP. In order to calculate the cross-covariance, we first divided each spike train into bins of $\Delta t = 1\,\mathrm{ms}$. The number of spikes of neuron i within the interval $(t,\, t + \Delta t)$ is represented by $n_i(t)$. The cross-covariance between the spike trains of neuron i and neuron j is calculated as:

$$CC_{ij}(t) = \frac{1}{T} \sum_{t'=1}^{T} \Big(n_i(t') - \langle n_i \rangle\Big) \Big(n_j(t' + t) - \langle n_j \rangle\Big), \tag{S14}$$

where T is the total number of bins, and $\langle n_i \rangle$ is the average of $n_i(t)$ over all bins. Finally, we averaged $CC_{ij}(t)$ across 100 randomly chosen neurons in the network (4950 pairs, figure S3). The results show that except for the cases where the excitatory-to-excitatory synaptic weights grow very large (figure S3, gray panels), the average cross-covariance of spike trains is indeed very close to zero, and the network remains in the uncorrelated state. As indicated in the article, in the cases where the baseline correlations are non-zero, our method fails to accurately predict the structures arising from STDP.
